# A computational model of 1,5‐AG dynamics during pregnancy

**DOI:** 10.14814/phy2.13375

**Published:** 2017-08-21

**Authors:** Seyedeh M. Zekavat, Slava Butkovich, Grace J. Young, David M. Nathan, Danny Petrasek

**Affiliations:** ^1^ Broad Institute of MIT and Harvard Cambridge Massachusetts; ^2^ Massachusetts Institute of Technology Cambridge Massachusetts; ^3^ California Institute of Technology Pasadena California; ^4^ Harvard Medical School Boston Massachusetts; ^5^ Massachussetts General Hospital Boston Massachusetts

**Keywords:** 1,5‐anhydroglucitol, computational model, diabetes, gestational diabetes, pregnancy

## Abstract

The importance of 1,5‐anhydroglucitol (1,5‐AG) as an intermediate biomarker for diabetic pregnancy is multi‐fold: (1) it serves as a reliable indicator of moderate‐level glycemic control, especially during early gestation; (2) it has been associated with increased risk of diabetes, independent of HbA1c and fasting glucose; and (3) it is an independent risk factor for the development of eclampsia during pregnancy. However, the clinical use of this biomarker during pregnancy has been underutilized due to physiological changes in glomerular filtration rate, plasma volume, and other hemodynamic parameters which have been hypothesized to bias gestational serum 1,5‐AG concentrations. Here, we develop an *in‐silico* model of gestational 1,5‐AG by combining pre‐existing physiological data in the literature with a two‐compartment mathematical model, building off of a previous kinetic model described by Stickle and Turk (1997) Am. J. Physiol., 273, E821. Our model quantitatively characterizes how renal and hemodynamic factors impact measured 1,5‐AG during normal pregnancy and during pregnancy with gestational diabetes and diabetes mellitus. During both normal and diabetic pregnancy, we find that a simple two‐compartment model of 1,5‐AG kinetics, with all parameters but reabsorption fraction adjusted for time in pregnancy, efficiently models 1,5‐AG kinetics throughout the first two trimesters. Allowing reabsorption fraction to decrease after 25 weeks permits parameters closer to expected physiological values during the last trimester. Our quantitative model of 1,5‐AG confirms the involvement of hypothesized renal and hemodynamic mechanisms during pregnancy, clarifying the expected trends in 1,5‐AG to aid clinical interpretation. Further research and data may elucidate biological changes during the third trimester that account for the drop in 1,5‐AG concentrations, and clarify physiological differences between diabetes subtypes during pregnancy.

## Introduction

The treatment and monitoring of diabetes relies on tight monitoring of glycemic control by measuring biomarkers in the body (The Diabetes Control and Complications Trial Research Group, 1993; UK Prospective Diabetes Study (UKPDS) Group, 1998a; UK Prospective Diabetes Study (UKPDS) Group, 1998b). Clinically, there are two common biomarkers that are used to measure glycemic control: daily glucose monitoring and HbA1c. Plasma glucose levels reflect an instantaneous, short‐term measure of glycemic control while HbA1c levels provide insight on long‐term glycemic control, averaged over 3–4 months (Buse et al. 2003). According to the Diabetes Control and Complications Trial group, mean HbA1c does not fully capture glycemic control since it is a long‐term measure; rather, “complications may be more highly dependent on the extent of postprandial glycemic excursions” (The Diabetes Control and Complications Trial Research Group, 1995). More recently, studies indicate that measures of intermediate‐term glycemic control, specifically those reflective of postprandial glucose fluctuations, are independent risk factors for the development of macrovascular complications (Hanefeld et al. 1996; Muggeo et al. 2000; Temelkova‐Kurktschiev et al. 2000; Dungan et al. 2006). Thus, used in parallel to short‐term and long‐term measures of glycemic control, an intermediate‐term biomarker that is indicative of postprandial glucose excursions may be useful in the management of patients with diabetes and informative of the risk of macrovascular complications.

The polyol 1,5‐anhydroglucitol (1,5‐AG), quantifiable in plasma and cerebrospinal fluid, is a molecule of significant interest in the assessment of diabetes mellitus that has been proposed as a marker for postprandial hyperglycemia. 1,5‐AG intake is mainly through ingestion (average of 5 mg daily) (McGill et al. 2004) and the major route of elimination is through urinary excretion, with intake and elimination at approximate equilibrium. Normally, 1,5‐AG is filtered in the kidneys and completely reabsorbed in the renal tubules back into the bloodstream (Buse et al. 2003), leading to a stable 1,5‐AG plasma concentration of ~ 20 mg/mL (Mehta et al. 2012). However, with elevated serum glucose concentrations (>180 μmol/L), plasma 1,5‐AG levels fall due to competitive inhibition of renal reabsorption by glucose. Thus, decreased plasma concentrations of 1,5‐AG are observed in diabetes, secondary to competitive tubular reabsorption of 1,5‐AG with hyperglycemia (Sermer et al. 1998). 1,5‐AG levels recover within 24–72 h posthyperglycemia (Yamanouchi et al. 1992, 1996), reflecting even transient elevations of glucose within the past few days and thereby serving as a measurement which is informative of glycemic excursions even in patients with normal HbA1cs (Kishimoto et al. 1995). Indeed, 1,5‐AG has been associated with risk of developing diabetes independently of HbA1c and fasting glucose concentrations (Juraschek et al. 2012).

**Table 1:** Summary of the model.

Parameter	Description	Units	Representation in model	Average standard deviation^a^	Implementation of Data	Data source
*t*_preg_	Time during pregnancy	Days	*t*_preg_	–	–	–
GFR (*t*_preg_)	Glomerular filtration rate	mL/day	$-0.077*{t_{\mathrm{preg}}}^{2}+4.17*t_{\mathrm{preg}}+99.04$	21.2	Used data to calculate fit in Figure 2 (*n* = 25, normal pregnancy)	Dunlop (1981)
*V*_plasma_ (*t*_preg_)	Plasma volume	mL	$\frac{796.2}{0.62+4.4*e^{\left(0.21*\left(t_{\mathrm{preg}}-9.2\right)\right)}}+2378$	52.3	Used data to calculate fit in Figure 2 (*n* = 52 to 68, normal pregnancy)	Whittaker and Lind (1993)
$f_{\mathrm{plasma}}^{1,5-\mathrm{AG}}\left(t_{\mathrm{preg}}\right)$	Fractional plasma mass	Unitless	−0.006*t_preg_+0.3	0.12	Used data to calculate fit in Figure 2 (*n* = 27, normal pregnancy)	Pipe et al. (1979)
*k*_*i*_	1,5‐AG input rate	mg/day	5	**–**	Used data in ODE	Stickle and Turk (1997)
[G]	Mean maximal glucose concentration	mM	$\left\{\begin{array}{c} <\;\;7.5\;\;\text{mM}\;\text{for}\;\;\text{normal}\;\;\text{pregnancies}\;\\ 8.9\;\;\text{mM}\;\;\text{for}\;\;\text{GDM/DM}\;\;\text{pregnancies}\; \end{array}\right.$	**–**	Used raw values in model	Nowak et al. (2013)
*r*([G])	Reabsorption fraction	Unitless	$\left\{\begin{array}{c} 0.9984\;\;\text{if}\;\;[G]\;\;<\;\;7.4\;\;\text{mM}\\ -\left(0.0026\;\;{\text{mM}}^{-1}\right)*\left[G\right]+1.018\;\;\text{if}\;\;\left[G\right]\;\;<\;\;7.4\;\;\text{mM} \end{array}\right.$	**–**	Used equation for *r* estimation	Stickle and Turk (1997)
r(*t*_preg_, [*G*])	Reabsorption fraction	Unitless	$\begin{array}{cc} & \text{For}\;\;\left[G\right]\;\;<\;\;7.4\;\;\text{mM}:\left\{\begin{array}{c} a\;\;\text{if}\;\;t_{\mathrm{preg}}\;\;\leq \;\;25\;\;\text{weeks}\\ a-b*\left(t_{\mathrm{preg}}-25\right)\;\;\text{if}\;\;t_{\mathrm{preg}}>25\;\;\text{weeks}\; \end{array}\right.\\ & \text{For}\;\;\left[G\right]\;\;>\;\;7.4\;\;\text{mM}:\left\{\begin{array}{c} a-c\left[G\right]\;\;\text{if}\;\;t_{\mathrm{preg}}\;\;\leq \;\;25\;\;\text{weeks}\\ a-b*\left(t_{\mathrm{preg}}-25\right)-c\left[G\right]\;\;\text{if}\;\;t_{\mathrm{preg}}>25\;\;\text{weeks}\; \end{array}\right. \end{array}$where *a* and *b* refer to best‐fitted values from Figure 4	**–**	Used 1,5‐AG data to estimate best‐fitted *a* and *b* for an alternative *r* estimation during pregnancy timeline	Tetsuo et al. (1990)

The stated parameters were used in an ordinary differential equation (ODE) to find 1,5‐AG levels. For each value of *t*_preg_ examined, the ODE was used to find the corresponding steady state level of 1,5‐AG. This information was compiled for many values of *t*_preg_ to provide an overall picture of how 1,5‐AG levels change throughout pregnancy.

a Average standard deviations across all available time points visualized in Figure 2 are reported for each parameter (for fractional plasma mass, the standard deviations are averaged for the three time points during pregnancy, excluding week 0).

While intermediate‐term biomarkers of glycemic control such as 1,5‐AG may be useful to monitor diabetes in the general population, they are even more critical in gestational diabetes mellitus (GDM) and diabetes mellitus (DM) during pregnancy — diseases complicating greater than 17% of pregnancies (Sacks et al. 2012). Due to the 3–4 month timeline by which HbA1c levels fluctuate, HbA1c does not optimally capture intermediate‐term glycemic excursions during the critical 9 month period of gestation. Although an automated enzymatic assay (Glycomark) has been approved in the U.S. to measure plasma 1,5‐AG levels (McGill et al. 2004), and a similar assay has been used in Japan for over two decades (Fukumura et al. 1994), some studies do not recommend monitoring 1,5‐AG for glycemic control during pregnancy, since physiological changes in glomerular filtration rate, plasma volume, and other hemodynamic parameters during gestation may bias 1,5‐AG concentrations in the blood (Kilpatrick et al. 1999; Tam et al. 2001). Nonetheless, several studies have suggested that 1,5‐AG concentrations during pregnancy are reliable indicators of moderate‐term glycemic control, especially during early stages of pregnancy (Yamanouchi et al. 1996; Buse et al. 2003; Dworacka et al. 2006; Andronesi et al. 2012; Boritza et al. 2014). Moreover, multiple studies have suggested postprandial glucose to be an independent risk factor for the development of pre‐eclampsia and eclampsia during later stages of pregnancy (Sermer et al. 1998; Rowan et al. 2010). Thus, while an intermediate‐term biomarker of hyperglycemic excursions may be clinically beneficial in informing risk and motivating improved monitoring and prevention of such vascular disease (Fukumura et al. 1994), a quantitative understanding of the physiological parameters impacting 1,5‐AG baseline dynamics during pregnancy has not been sufficient to allow transformation of 1,5‐AG into a meaningful parameter in the context of the renal and hemodynamic changes that occur in pregnancy.

Here, we develop an *in‐silico* model of 1,5‐AG during pregnancy. By using published data and applying it to a two‐compartment mathematical model, we are attempting to more precisely characterize 1,5‐AG physiological behavior in the context of renal and hemodynamic parameters for the monitoring of GDM and DM during pregnancy. We use the original compartment model for 1,5‐AG by Stickle and Turk (Stickle and Turk 1997) and adapt the model for diabetes in pregnant populations by accounting for physiologic changes in GFR, reabsorption fraction, plasma volume, and fractional plasma mass. One limitation of the study is the paucity of available data on GFR, plasma volume, and fractional plasma mass; as such, we were unable to distinguish between normal individuals and different diabetic subtypes with respect to these baseline hemodynamic and renal parameters during the gestational timeline. We hope that providing a mathematical context can shed light onto the physiological mechanisms that have been hypothesized to impact gestational 1,5‐AG dynamics and motivate further research to improve understanding of 1,5‐AG during pregnancy, decreasing the barrier to clinical use of this biomarker.

## Methods

### Compartment model for 1,5‐Anhydroglucitol

**Figure 1:**
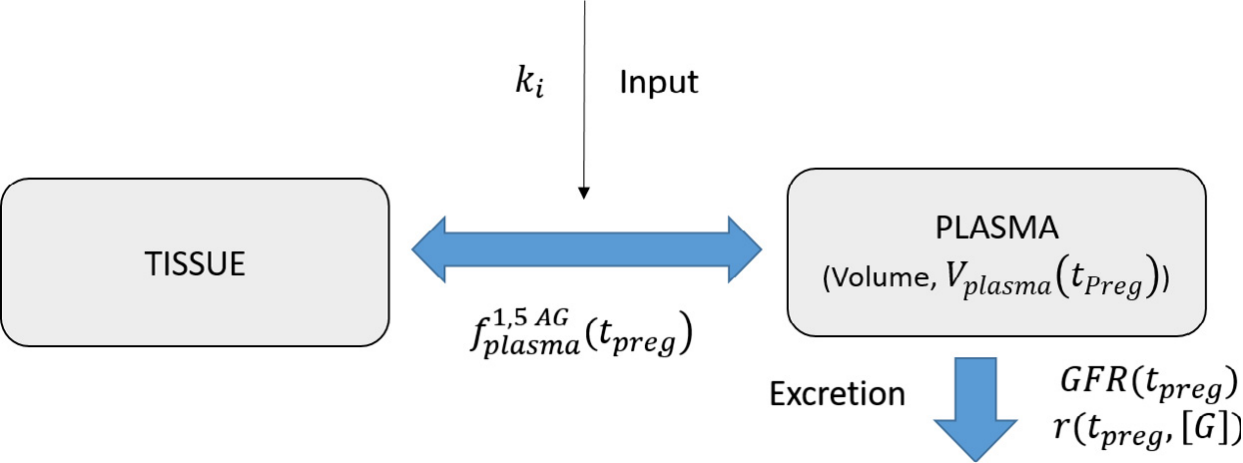
Compartment model for 1,5‐AG. This two‐compartment model describes 1,5‐AG dynamics in plasma and tissue over timeline in pregnancy (*t*_preg_), assuming rapid partitioning between tissue and plasma pools. The fraction of 1,5‐AG in the plasma is proportional to the fraction of plasma mass to total mass in a given individual. For pregnant individuals, we model this as a fraction that varies with time in pregnancy $\left(f_{\mathrm{plasma}}^{1,5-\text{AG}}\left(t_{\mathrm{preg}}\right)\right)$ and is approximated to be independent of glycemic state. The 1,5‐AG input rate, *k*_*i*_, represents both ingestion and endogenous production of 1,5‐AG. 1,5‐AG is excreted from plasma through the kidneys, and the rate at which it does so is dependent on both the glomerular filtration rate, modeled as a function of *t*_preg_, (*GFR(t*_preg_ *)*), and reabsorption in the glomerular tubules, modeled as a function of *t*_preg_ and glucose concentration, (*r(t*_preg_ *, [G])*). For purposes of dimensional analysis, *k*_*i*_ and GFR were scaled by plasma volume (*V*_plasma_(*t*_preg_)). See equation 1 and Table 1 for additional details.

Steady‐state two‐compartment (plasma and tissue) mass balance models for the presence of 1,5‐AG in plasma have been validated by Yamanouchi et al. (1991) and Stickle and Turk (1997). Here, we describe an extension of this two‐compartment model towards normal pregnancy and toward pregnancy with diabetes. As previously noted, the paucity of existing data limits our ability to distinguish between different diabetic subtypes during pregnancy. Our two‐compartment model (Fig. 1) describes 1,5‐AG dynamics in plasma and tissue over timeline in pregnancy *t*_preg_, assuming rapid partitioning between plasma and tissue pools. We describe the concentration of 1,5‐AG in plasma over time (*t*, representing time to steady‐state) and over timeline in pregnancy (*t*_preg_), as in the differential equation in equation 1 below. For the purposes of plotting $C_{\mathrm{plasma}}^{1,5-\mathrm{AG}}$ as a function of *t*_*preg*_ in the paper, we assume steady‐state levels of $C_{\mathrm{plasma}}^{1,5-\mathrm{AG}}$ as a function of *t*, which is physiologically relevant for individuals.

(1) $$\frac{dC_{\mathrm{plasma}}^{1,5-\mathrm{AG}}}{dt}=f_{\mathrm{plasma}}^{1,5-\mathrm{AG}}\left(t_{\mathrm{preg}}\right)*\left[\frac{k_{i}}{V_{\mathrm{plasma}}\left(t_{\mathrm{preg}}\right)}-\text{GFR}\left(t_{\mathrm{preg}}\right)*\frac{C_{\mathrm{plasma}}^{1,5-\mathrm{AG}}}{V_{\mathrm{plasma}}\left(t_{\mathrm{preg}}\right)}*\left(1-r(t_{\mathrm{preg}},\left[G\right])\right)\right]$$

In equation 1, 1,5‐AG plasma input rate is assigned as *k*_*i*_ (representing both ingestion and endogenous production of 1,5‐AG) and is balanced in the steady state by excretion rate through the kidneys. As seen in Table 1, *k*_*i*_ is approximated as 5 mg/day from Yamanouchi et al. (1991), who approximated 1,5‐AG supplement through foods as 4.38 mg/day on eight healthy subjects, mean urinary 1,5‐AG excretion as 4.76 mg/day (with negligible excretion into stools), and 0.4 mg/day of *de novo* synthesis of 1,5‐AG suggested in patients without oral supplement of 1,5‐AG.

1,5‐AG is rapidly repartitioned between plasma and tissue pools, with the fraction of 1,5‐AG in the plasma being proportional to the fraction of plasma mass to total mass in a given individual. For pregnant individuals, we model this as a fraction which varies with time in pregnancy $\left(f_{\mathrm{plasma}}^{1,5-\mathrm{AG}}\left(t_{\mathrm{preg}}\right)\right)$ and is approximated to be independent of glycemic state (Whittaker and Lind 1993).

1,5‐AG is excreted from plasma through the kidneys, and the rate at which it does so is dependent on both the glomerular filtration rate (GFR) and on reabsorption in the glomerular tubules (*r*). As found via patient data, glomerular filtration rate changes substantially throughout pregnancy and is modeled as a function of time in pregnancy, *GFR*(*t*_preg_) described in the results.

High glucose concentrations inhibit reabsorption of 1,5‐AG and increase the excretion rate, leading to net depletion of plasma 1,5‐AG. This phenomenon has been modeled by Stickle and Turk (1997) via equation 2 below, describing reabsorption fraction at normal and high glucose concentrations:

(2) $$r\left\{\begin{array}{c} 0.998\;\;\text{if}\;[G]<7.4\;\text{mM}\\ -\left(0.0026\;{\text{mM}}^{-1}\right)*\left[G\right]+1.018\;\;\text{if}\;\left[G\right]>7.4\;\text{mM} \end{array}\right.$$

As seen in Figure 1 and later in Figure 4, we adapt this model by allowing for the incorporation of time in pregnancy into *r*:*r*(*t*_preg_, [*G*]). While glucose concentrations do fluctuate over the time‐span of minutes in both normal and diabetic individuals, as shown by Stickle et al. (via their Fig. 2), this short‐term fluctuation does not significantly alter 1,5‐AG levels in normal or diabetic individuals.

**Figure 2:**
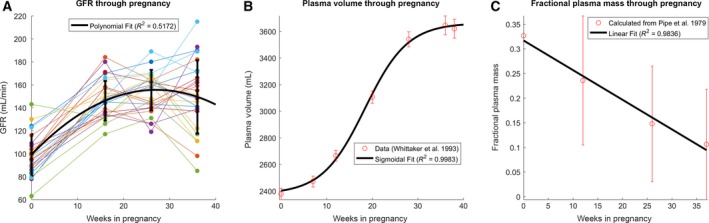
Fitting physiological parameters throughout pregnancy. These fits were used in our model for 1,5‐AG during normal pregnancy. (A) Glomerular filtration rate (GFR), using data from 25 nondiabetic pregnant individuals in Dunlop 1981 and fitting to a second‐order polynomial (eq. 3). (B) Plasma volume, using longitudinal data from approximately 69 healthy females throughout seven times in pregnancy (Sacks et al. 2012) and fitting to a sigmoidal curve (eq. 4). (C) Fractional plasma mass, using data from 27 nondiabetic pregnant individuals in Pipe et al. 1979 and fitting to a line (eq. 6). This value was found by dividing the increase in plasma mass at each time point by the total body mass gained, since the start of pregnancy (eq. (5)). Average standard deviations across all available time points visualized in Figure 2 are reported for each parameter (for fractional plasma mass, the standard deviations are averaged for the three time points during pregnancy, excluding week 0).

As will be shown, the steady‐state mass balance model proposed by Yamanouchi and Akanuma (1994) and Stickle and Turk (1997) can be expanded to include the kinetic characteristics of 1,5‐AG behavior during diabetic pregnancy. The implications of the kinetic model for the use of 1,5‐AG monitoring in the evaluation of glycemic control are discussed.

### Fitting physiological data from the literature

The literature was explored for relevant physiological data points that would allow us to adapt the pre‐established model for cases of normal pregnancy and diabetic pregnancy (GDM/DM). Parameters included factors that were suggested to strongly affect 1,5‐AG concentration in plasma including GFR, fraction of plasma to tissue mass, and plasma volume; value change versus time during gestational progression was noted. GFR data for nondiabetic and diabetic pregnancies were derived from a longitudinal study by Dunlop (1981) which examined 25 healthy women during pregnancy using inulin clearance. Plasma volume for normal pregnancy was derived from Whittaker and Lind (1993), a longitudinal prospective study examining albumin concentration and plasma volume in 69 healthy pregnant women. Fraction of plasma to total body mass $\left(f_{\mathrm{plasma}}^{1,5-\mathrm{AG}}\left(t_{\mathrm{preg}}\right)\right)$ was derived using data from an investigation by Pipe et al. (1979) which longitudinally examined 27 women during normal pregnancy utilizing body composition analysis. Due to the limited availability of data, we were unable to differentiate between normal pregnancy and different subtypes of diabetic pregnancy (GDM/DM) with respect to GFR, plasma volume, and fraction of plasma to tissue mass.

These data were used to find fits over time in pregnancy for GFR, plasma volume, and fraction of mass from plasma using least squares fitting. All error bars represent one standard deviation. In calculating the error bars for fractional plasma mass, we first calculated the standard deviation of the increase in total body weight for each time point; the error bars illustrate the reciprocals of these values. Standard deviations for plasma mass were ignored as they were not provided in Pipe et al. (1979).

1,5‐AG data across weeks of gestation from normal pregnant individuals and pregnant individuals with GDM/DM were acquired from a longitudinal study by Tetsuo et al. (1990), which evaluated serum concentrations of 1,5‐AG in 543 normal pregnant women and 75 pregnant women with GDM/DM. These data were used to compare the mathematical model to observed 1,5‐AG measurements.

### Computational modeling & parameter derivation

The fits from the literature data were utilized as parameters in our ODE system (eq. 1). The ODE was solved using the ode15s differential equation solver in MATLAB (Natick, MA). In fitting the ODE to 1,5‐AG data, we minimized the square of the difference using a termination tolerance for the function value of 1e‐5.

## Results

### Fitting physiological data from the literature

We mined published data for parameters that were suggested to strongly affect 1,5‐AG concentration in plasma during the gestational period, including GFR, plasma volume, and fraction of plasma to tissue mass, and found best fit equations that modeled their change over gestational time.

To find an expression that adequately fit the overall GFR data, the individual data series for 25 nondiabetic patients across four time points (before pregnancy, 16 weeks pregnant, 26 weeks pregnant, and 36 weeks pregnant) were examined (Dunlop 1981). For most patients, the GFR trajectory followed a parabolic path explained by a best‐fit equation (eq. 3) (*R* ^2^ = 0.52), increasing by a maximum of 1.57‐fold, with a peak at time 27.1 weeks in pregnancy.

(3) $$\text{GFR}=-0.077*{t_{\mathrm{preg}}}^{2}+4.17*t_{\mathrm{preg}}+99.04\;\left[\text{mL}/\text{min}\right]$$

The plasma volume throughout pregnancy was fit with a sigmoidal curve (eq. 4) (*R* ^2^ = 0.998).

(4) $$\frac{796.2}{0.62+4.4*e^{\left(0.21*\left(t_{\mathrm{preg}}-9.2\right)\right)}}+2378\;\left[\text{mL}\right]$$

The fractional plasma mass represents the fraction of mass gained since the start of pregnancy attributable to plasma gain. The increase in plasma mass ($\Delta M_{\mathrm{plasma}}$) and increase in total body mass ($\Delta M_{\mathrm{total}}$) since the start of pregnancy were derived using data from Pipe et al. (1979). The fractional plasma mass was calculated by finding the ratio between the former and the latter (eq. (5)). These calculated values were then fitted to a linear expression with respect to time in pregnancy (eq. 6) (*R* ^2^ = 0.98).

(5) $$f_{\mathrm{plasma}}^{1,5-\mathrm{AG}}=\frac{\Delta M_{\mathrm{plasma}}}{\Delta M_{\mathrm{total}}}$$

(6) $$f_{\mathrm{plasma}}^{1,5-\mathrm{AG}}=-0.006*t_{\mathrm{preg}}+0.32$$

### Modeling 1,5‐Anhydroglucitol

**Figure 3:**
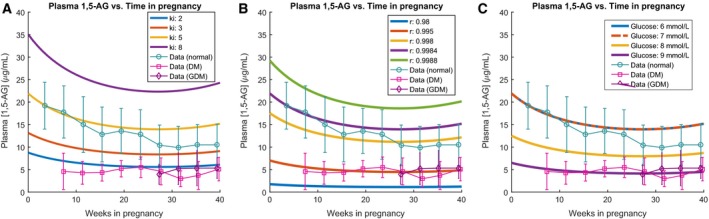
Sensitivity analysis over parameters in the 1,5‐AG model. In each plot, one parameter was changed while the others were held constant. Superimposed data over time are from Tetsuo et al. 1990 (A) Iterations over *k*_*i*_, keeping the reabsorption fraction (*r*) at 0.9984. (B) Iterations over *r*, keeping *k*_*i*_ at 5. (C) Iterations over mean maximal glucose levels, keeping *k*_*i*_ at 5. The reabsorption fraction was calculated using equation 2.

Using the fits over time in pregnancy for GFR, plasma volume, and fractional plasma mass, we used an ordinary differential equation (eq. 1) to model the plasma concentration of 1,5‐AG throughout timeline of pregnancy. Before fitting all parameters in the model simultaneously to the normal and diabetic (GDM/DM) pregnancy timeline 1,5‐AG data, we first wanted to understand how each of the main individual parameters *r*,* k*_*i*_, and [*G*] separately affected plasma 1,5‐AG levels throughout gestation. Thus, we performed sensitivity analysis, iterating across these three parameters individually while keeping all other parameters constant (Fig. 3). The modeled 1,5‐AG results were superimposed over points derived from healthy, GDM, and DM patients (Tetsuo et al. 1990) to provide insight on the individual effect of each parameter on 1,5‐AG levels.

Figure 3A, B, and C show the behavior of the model across different fold changes of parameters *k*_*i*_, *r*, and [*G*], respectively. Since *k*_*i*_ represents the input rate of 1,5‐AG into the body, we expect that increased *k*_*i*_ co‐occurs with higher 1,5‐AG levels in plasma. This matches what is observed in Figure 3A, where a range of *k*_*i*_ values were examined while keeping the reabsorption fraction constant at 0.9984. We see that scaling *k*_*i*_ similarly scales 1,5‐AG levels, with a fourfold increase in *k*_*i*_ yielding approximately a fourfold increase in 1,5‐AG, as expected from the linear relationship between 1,5‐AG and *k*_*i*_ from equation 1. A *k*_*i*_ of 5 yields a plot that stays within one standard deviation of the 1,5‐AG data for normal patients, while a *k*_*i*_ of 2 yields a plot that stays within one standard deviation of the 1,5‐AG data for DM patients. However, we would expect the dietary 1,5‐AG input levels to be roughly the same between normal, DM, and GDM patients (although some research suggests that 1,5‐AG levels may be sensitive to dairy intake) (Koga et al. 2010); therefore, we other physiological factors aside from *k*_*i*_ are likely causing this difference.

We then moved on to examine how changes in the reabsorption fraction could affect the model, keeping *k*_*i*_ at a constant nominal value of 5. As expected, higher reabsorption fraction corresponds to higher plasma 1,5‐AG values, as seen in Figure 3B. One observation to note is that small differences in the reabsorption fraction lead to large differences at the concentration of 1,5‐AG; we see that an increase in *r* by 0.003 yields a two‐ to threefold increase in 1,5‐AG levels, which can correspond to the difference between a pregnant diabetic patient and a normal patient. Values of *r* between approximately 0.998 and 0.9984 correspond to the normal case, while a value of 0.995 corresponds to the pregnant diabetic case.

We wished to directly examine the effect of glucose on 1,5‐AG levels through implicit changes in the reabsorption fraction. From equation 2 we see that for all glucose concentrations below 7.4 mmol/L, the reabsorption fraction stays at a constant value of 0.9984, and as plasma glucose concentration rises above 7.4 mmol/L, the reabsorption fraction linearly decreases with glucose concentration. We performed iterations on the mean maximal glucose concentration, keeping *k*_*i*_ at a constant value of 5 (reflecting nominal *k*_*i*_ in healthy individuals, derived from Stickle and Turk (1997)). We would predict that as glucose concentration increases above 7.4 mmol/L, 1,5‐AG plasma concentration decreases due to decreased reabsorption fraction. This prediction matches the results we see in Figure 3C. Glucose levels below 7.4 mmol/L fall within the range expected for nondiabetic patients, based on the superimposed data from Tetsuo et al. (1990). We see that a glucose level of 9 mmol/L corresponds closely with the data from pregnant diabetic individuals. Thus, differences in glucose concentration – through their effect on the reabsorption fraction – seem to have a large effect on plasma 1,5‐AG concentrations. As expected, pregnant diabetic individuals have higher plasma glucose concentrations than normal individuals, which correspond to smaller reabsorption fractions and thus lower 1,5‐AG levels.

**Figure 4:**
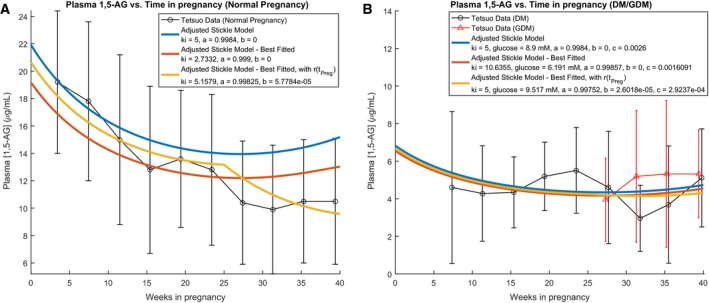
Modifications to the model. Both the normal (A) and the pregnant diabetic cases (B) are considered separately. Each case illustrates three lines: The blue lines represent the Adjusted Stickle Model, or equation 1 using nominal parameters for *k*_*i*_ and *a* from Stickle and Turk 1997 and adjusting by *t*_*P*__*reg*_ for GFR, fractional plasma mass, and plasma volume, while assuming that *r* is constant with respect to *t*_*P*__*reg*_ (i.e., b = 0, from eq. 7). The red lines represent the Best Fitted Adjusted Stickle Model, or the Adjusted Stickle Model using least squares fitted values for *k*_*i*_ and *a*, with b = 0. The yellow lines represent modifications of the Best Fitted Adjusted Stickle Model, additionally using least squares fitted values for b. (A) Normal pregnancy. (B) Diabetic pregnancy. The Adjusted Stickle Model uses [G] = 8.9 mM as a representative value for the mean maximal glucose concentration in diabetic patients, and *k*_*i*_ = 5 at a fixed parameter. The fitted models have *r* = *a*‐b* (*t*_preg_‐25) –*c* * [*G*].

We further sought to investigate our complete model across combinations of physiological parameters. In Figure 4A, we examine 1,5‐AG levels for normal pregnancy. The blue line represents the Adjusted Stickle Model, which illustrates the model for the default values of the system (*k*_*i*_ = 5, *r* calculated using eq. 2). For normal pregnancy, it is assumed that the glucose level is low enough such that *r* stays constant at 0.9984. The red line (Best Fitted Adjusted Stickle Model) represents our model with the values of *k*_*i*_ and *r* best‐fitted to the 1,5‐AG data. The Adjusted Stickle Model deviates from the mean 1,5‐AG data points around 25 weeks, while the Best Fitted Adjusted Stickle Model has a low *k*_*i*_ (2.73 compared to the expected 5) and a high *r* (0.999 instead of the expected 0.9984). This motivated the re‐evaluation of the model with respect to time in pregnancy, and in particular evaluating the one parameter that did not depend on *t*_preg_ in the Adjusted Stickle Model, *r*. Using a function of *r* that depends on *t*_preg_ as in equation 7 (corresponding to the yellow line in Fig. 4A), where b is equal to 0 before 25 weeks and nonzero after 25 weeks, allows for not only improved fitting of the pregnancy data, but also physiological values much closer to those used in Stickle and Turk (1997), with *k*_*i*_ = 5.16, *r* = 0.99825 before 25 weeks, and *r* = 0.99825‐5.78*10^−5^*(*t*_preg_‐25) after 25 weeks. This result suggests that there may be physiological changes during the third trimester of pregnancy that decrease the reabsorption fraction.

(7) $$r=\left\{\begin{array}{c} a\;\mathrm{if}\;t_{\mathrm{preg}}\leq 25\;\text{weeks}\\ a-b*\left(t_{\mathrm{preg}}-25\right)\;\;\text{if}\;t_{\mathrm{preg}}>25\;\text{weeks} \end{array}\right.$$

We then performed analogous analyses to the pregnant diabetic case, using 1,5‐AG data from untreated DM and GDM patients from Tetsuo et al. (1990). For the unfitted model (Adjusted Stickle), we used a glucose concentration of 8.9 mmol/L, from the mean maximal glucose concentration of third trimester type 1 diabetic subjects in an analysis by Nowak et al. (2013), as we expected the mean maximal glucose level of an untreated pregnant diabetic patient to be higher than that for a nondiabetic patient. In the Best Fitted Adjusted Stickle Model, where *r* is not a function of *t*_preg_ (red line in plot), the fitted *k*_*i*_ value is high (10.64 compared to the expected 5), and the best‐fitted glucose concentration is low (6.19 mmol/L compared to the expected ~8.9 mmol/L). For the yellow line (Best Fitted Adjusted Stickle Model, with *r*(*t*_preg_), in addition to having *r* depend on *t*_preg_, we also fixed the value of *k*_*i*_ at 5, assuming that physiologically, *k*_*i*_ will not significantly differ between pregnant diabetic and nondiabetic subjects. Doing this, we get a fitted glucose value of 9.5 mmol/L, which is higher than our original value of 8.9 mmol/L but is still within one standard deviation of the mean maximal glucose levels of diabetics in both second and third trimesters of pregnancy (Nowak et al. 2013).

For both the normal and GDM cases, we see that finding the best fit of the model while allowing *r* to depend on *t*_preg_ after 25 weeks allows us to achieve parameters closer to expected physiological values.

## Discussion

Given the limited analysis of quantitative physiology on 1,5‐AG during gestation, the present analysis sought to create a computational model to better guide interpretation of measured 1,5‐AG in both normal and diabetic pregnancy. Stickle and Turk (1997) model was recalibrated for the dynamic biological parameters of gestation in an attempt to better define the variables affecting changes in 1,5‐AG during gestation. The purpose of this model is to provide a framework to computationally simulate the major mechanisms affecting 1,5‐AG in human pregnancy. To the best of our knowledge, we are the first in the literature to synthesize data from multiple sources and computationally analyze the mechanisms perturbing 1,5‐AG levels during pregnancy.

Herein, we mathematically model 1,5‐AG fluctuations during pregnancy by utilizing the steady‐state solution of a differential equation combined with clinical values from the literature. Three widely cited physiologic variables fluctuating during pregnancy include GFR, plasma volume, and fractional plasma mass (Pitkin 1976; Pipe et al. 1979; Dunlop 1981; Ezimokhai et al. 1981; Whittaker and Lind 1993; Krzyzanowska et al. 2008). A second‐order polynomial was fitted to GFR, while a sigmoidal curve was fitted to plasma volume. Fraction of plasma to tissue mass – representing the fraction of mass gained during pregnancy due to an increase in plasma volume – were not explicitly found in the literature, and were extrapolated utilizing other data points and found to linearly increase with time in pregnancy (Pipe et al. 1979; Whittaker and Lind 1993). The best‐fit equations were subsequently used in equation 1 to simulate 1,5‐AG and compare with 1,5‐AG data in normal and diabetic pregnancies from the literature (Koga et al. 2010). Limitations of this methodology include low sample sizes from the population, since the 1990 study by Tetsuo et al. (1990) is the only comprehensive study which measured serum 1,5‐AG levels during pregnancy in normal and diabetic females. Thus, additional clinical data regarding 1,5‐AG levels during pregnancy would need to be available for cross‐validation of such a model.

In normal pregnancy, a tight fit was found for <25 weeks using the same baseline parameters (*k*_*i*_, *r*) as in the Stickle Model and adjusting for parameters that varied with timeline in pregnancy (GFR, *V*_plasma_, $f_{\mathrm{plasma}}^{1,5-\mathrm{AG}}$); however, a deviation was seen from the model after 25 weeks (Adjusted Stickle Model in Fig. 4A). Furthermore, best‐fitting this model (Best Fitted Adjusted Stickle Model in Fig. 4A) for *k*_*i*_ and *r* results in nonphysiological parameters. However, upon allowing reabsorption fraction to vary with *t*_preg_ after 25 weeks of pregnancy as in equation 7 (Best Fitted Adjusted Stickle Model with *r*(*t*_preg_) in Fig. 4A), we find that not only do we arrive at physiological values for *k*_*i*_ and *r*, but we also acquire a better fit to the 1,5‐AG data. Similarly, in diabetic pregnancy (Fig. 4B), the Best Fitted Adjusted Stickle Model with *r*(*t*_preg_) using a fixed physiological *k*_*i*_ = 5, results in a best‐fit glucose concentration within 1 SD of the postprandial glucose concentration for pregnant diabetic cohorts found in the literature (Nowak et al. 2013). Furthermore, this model also results in expected best‐fit main effects for *r* (*a *=* *0.997), and expected relation of reabsorption fraction with [*G*] (*r* ~ 0.00029 * [*G*]), similar to that found in diabetics in Stickle and Turk (1997) (eq. 2). Thus, our mathematical model not only gives us the understanding that 1,5‐AG in nondiabetic and diabetic pregnancy can be simulated by the two‐compartment model with very little adjustment prior to 25 weeks, but that even minor alterations such as making reabsorption fraction a function of *t*_preg_ after week 25 can achieve a better fit while resulting in physiological parameters.

One of the insights from our findings is the potential presence of physiological changes during the third trimester that may affect the reabsorption fraction. Reabsorption rate may change as a function of time during the third trimester via biological mechanisms such as: (1) protein composition in the nephron (Cheung and Lafayette 2013), (2) changes in endogenous production of 1,5‐AG (Tetsuo et al. 1990), (3) shunting of 1,5‐AG to fetus (Boer et al. 2014), and (4) higher insulin requirements during late pregnancy (Davidson et al. 2016). Future studies may invest in acquiring additional longitudinal patient data across renal, hemodynamic, glycemic, and 1,5‐AG in individuals with GDM and DM during pregnancy to better model 1,5‐AG during the full nine‐month course of gestation.

## Conclusion

1,5‐AG is an informative indicator of intermediate‐term glycemic control especially during the early stages of gestation (Yamanouchi et al. 1996; Buse et al. 2003; Dworacka et al. 2006; Andronesi et al. 2012; Boritza et al. 2014), a risk factor for the development of eclampsia (Dunlop 1981; Mehta et al. 2012), and has been associated with altered risk of diabetes independent of HbA1c and fasting glucose (Fukumura et al. 1994; Hanefeld et al. 1996; Saito et al. 2014). The purpose of our model is to clarify how physiological changes during pregnancy (via GFR, reabsorption, plasma volume, and fractional plasma mass) affect 1,5‐AG level during gestation, building off of a previous kinetic model for nonpregnant individuals proposed by Stickle and Turk (1997). The impact of renal and hemodynamic parameters on 1,5‐AG during the gestational timeline has only been hypothesized in the literature; here we provide a mathematical support of such physiological factors, motivating improved clinical use and interpretation of 1,5‐AG levels during pregnancy.

Encouragingly, we find that a simple two compartment model of 1,5‐AG kinetics, with all parameters but reabsorption fraction adjusted for time in pregnancy, effectively models 1,5‐AG kinetics throughout the first two trimesters of pregnancy. In particular, we show that the decrease in mean 1,5‐AG levels during normal pregnancy is an expected result of hemodynamic changes, and that the fairly consistent and low 1,5‐AG levels in diabetic pregnancy are a result of altered intermediate‐term glycemic control. In addition to implementing the basic recommendations from Stickle and Turk's kinetic model, we find that making reabsorption a function of time in pregnancy in the last trimester was able to reconcile the 25‐week divergence of the original adjusted model from the patient data in normal gestation. The visual provided in Figure 4 may be used to clarify the expected trends in 1,5‐AG resulting from hemodynamic changes, aiding in clinical interpretation of 1,5‐AG levels during the course of pregnancy.

We hope that our mathematical approach can serve as a preliminary model of 1,5‐AG dynamics to motivate additional investigational research and patient data on the factors impacting 1,5‐AG during pregnancy. Quantitative models such as this may motivate improved clinical use and interpretation of 1,5‐AG levels during pregnancy.

## Conflict of Interest

The authors have no relevant affiliations or obligations to any organization or financial entity with respect to the research, authorship, and publication of this study.
